# A potent neutralizing antibody provides protection against SARS-CoV-2 Omicron and Delta variants via nasal delivery

**DOI:** 10.1038/s41392-022-01135-3

**Published:** 2022-08-30

**Authors:** Xinghai Zhang, Huajun Zhang, Tingting Li, Shaohong Chen, Feiyang Luo, Junhui Zhou, Peiyi Zheng, Shuyi Song, Yan Wu, Tengchuan Jin, Ni Tang, Aishun Jin, Chengyong Yang, Guofeng Cheng, Rui Gong, Sandra Chiu, Ailong Huang

**Affiliations:** 1 CAS Key Laboratory of Special Pathogens and Biosafety, Wuhan Institute of Virology, Center for Biosafety Mega-Science, Chinese Academy of Sciences, Wuhan, 430071 Hubei China; 2 Department of Immunology, College of Basic Medicine, Chongqing Medical University, Chongqing, 400010 China; 3 Chongqing Key Laboratory of Basic and Translational Research of Tumor Immunology, Chongqing Medical University, Chongqing, 400010 China; 4 University of Chinese Academy of Sciences, 100049 Beijing, China; 5 Division of Life Sciences and Medicine, University of Science and Technology of China, Hefei, 230026 Anhui China; 6 Key Laboratory of Molecular Biology on Infectious Diseases, Ministry of Education, Chongqing Medical University, Chongqing, 400010 China; 7 Mindao Haoyue Co., Ltd., SQ0042 Lanyuan Hall, No.61 Daxuecheng Middle Road, Chongqing, 400042 China

**Keywords:** Immunotherapy, Infectious diseases

## Abstract

Severe acute respiratory syndrome coronavirus 2 (SARS-CoV-2) is still rapidly spreading worldwide. Many drugs and vaccines have been approved for clinical use show efficacy in the treatment and prevention of SARS-CoV-2 infections. However, the emergence of SARS-CoV-2 variants of concern (VOCs), such as Delta (B.1.617.2) and the recently emerged Omicron (B.1.1.529), has seriously challenged the application of current therapeutics. Therefore, there is still a pressing need for identification of new broad-spectrum antivirals. Here, we further characterized a human antibody (58G6), which we previously isolated from a patient, with a broadly authentic virus-neutralizing activity that inhibits the Delta and Omicron variants with half-maximal inhibitory concentrations (IC_50_) of 1.69 ng/ml and 54.31 ng/ml, respectively. 58G6 shows prophylactic and therapeutic efficacy in hamsters challenged with the Delta and Omicron variants through nasal delivery. Notably, a very low dosage (2 mg/kg daily) of 58G6 efficiently prevented Omicron variant replication in the lungs. These advantages may overcome the efficacy limitation of currently approved neutralizing antibodies that can be administered only by intravenous injection. In general, 58G6 is a promising prophylactic and therapeutic candidate against current circulating VOCs and even future emerging mutants. To the best of our knowledge, 58G6 is one of the most potent neutralizing antibodies against Omicron, with a broader spectrum than those approved for clinical use. 58G6 could be developed as a nebulized therapy, which would be more cost effective and user friendly and enhance the clinical outcome compared to that obtained with direct nasal delivery.

## Introduction

COVID-19 is caused by severe acute respiratory syndrome coronavirus 2 (SARS-CoV-2), which has been circulating for over two years, causing severe respiratory illnesses and several millions of deaths worldwide.^1^ To contain this pandemic, efficient drugs and vaccines are highly desired. Drugs that can be developed include neutralizing monoclonal antibodies (nmAbs), small molecules and other inhibitors.^2,3^ Currently, a panel of nmAbs has been authorized for clinical use, and this formulation accounts for the majority of approved therapeutics. However, it has become apparent that SARS-CoV-2 can rapidly mutate its spike protein (S protein), which results in the generation of variants that could escape from immune-based treatment modalities, such as nmAbs. Public concern has been raised over the rapid spread of SARS-CoV-2 variants of concern (VOCs), including the Delta variant (B.1.617.2).^4^ Unfortunately, the Omicron variant (e.g., B.1.1.529) strikingly reduces the efficacy of most identified nmAbs^5–7^ due to its numerous mutations, including more than 30 genetic mutations in the S protein (https://www.who.int/publications/m/item/update-70-update-on-sars-cov-2-variant-of-concern-Omicron). It has spread rapidly and has overtaken the Delta variant, becoming the dominant strain globally.^8^ In addition, all currently approved nmAbs can be administered only by intravenous injection, which leads to the requirement of an extremely high dose to ensure their efficacies in vivo and a subsequent increase in patient burden. Therefore, the identification of antibodies with a broader antiviral spectrum, high efficiency and user-friendliness is highly desired and urgently needed.

Previously, 58G6, a potent nmAb, was isolated from recovered patients after SARS-CoV-2 infection and could neutralize the authentic original wild-type SARS-CoV-2 strain (SARS-CoV-2 WT) and the Alpha (B.1.1.7) and Beta (B.1.351) variants.^9^ In addition, it prevented body weight loss and reduced viral load in human ACE2 receptor-transgenic mice when given intraperitoneally. Since concerns have been rising regarding the loss of efficacy of approved nmAbs and vaccines due to the emergence of the Delta variant followed by the Omicron variant, we further evaluated 58G6 in vitro in terms of its neutralizing ability against more VOCs, including the Delta variant and the recently emerged Omicron variant. Notably, it potently inhibits the authentic Omicron variant with a half-maximal inhibitory concentration (IC_50_) of 54.31 ng/ml (subnanomolar range) in vitro. Additionally, we also evaluated its protective efficacy delivered via intranasal administration in a Syrian golden hamster model against the Delta and Omicron variants. When delivered via the respiratory tract (intranasal route), very low doses of 58G6 could protect hamsters from infection with the virus in vivo. In conclusion, 58G6 is a beneficial antiviral agent for the prevention of SARS-CoV-2 infection and the treatment of COVID-19 caused by all currently identified VOCs and future VOCs.

## Results

### 58G6 maintains binding to RBDs from SARS-CoV-2 WT and its variants

**Fig. 1:**
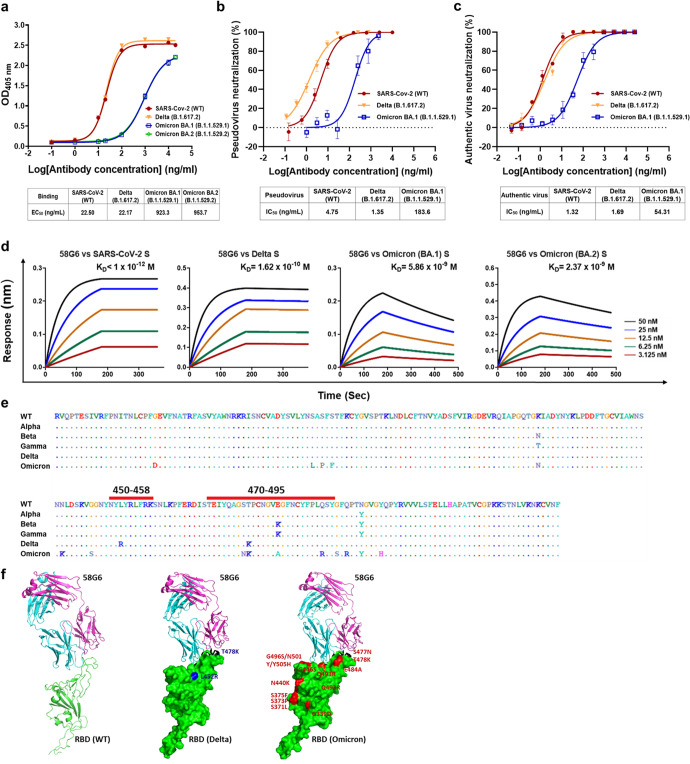
Activities of 58G6. **a** Binding of 58G6 to different RBDs, including SARS-CoV-2 WT, Delta, Omcrion BA.1 and BA.2, as measured by ELISA. **b** Neutralization of pseudotyped SARS-CoV-2 WT, Delta and Omcrion BA.1 by 58G6. Inhibitory rates of infection were calculated by scanning fluorescent plaques. **c** Evaluation of the neutralization activity of 58G6 against live SARS-CoV-2 WT and the Delta and Omicron variants. **d** The affinities of 58G6 for S proteins of SARS-CoV-2, Delta, Omicron BA.1 and Omicron BA.2 measured by BLI. **e** Binding regions of 58G6. RBDs from WT SARS-CoV-2 and the Alpha, Beta, Gamma, Delta and Omicron variants are aligned. The red lines indicate the binding regions (450–458, 470–495) of 58G6. **f** Structure of the complex of 58G6 Fab bound onto the SARS-CoV-2 RBD. On the left, the secondary structure elements of the RBD and heavy and light chains of 58G6 Fab are colored green, cyan and violet, respectively. The surface of the RBD is colored green. The mutated residues of the Delta variant are colored blue (middle), while those from the Omicron are colored red (right)

The major target of neutralizing antibodies and vaccines against SARS-CoV-2 is the S protein, which is the main antigenic component.^10^ To enter the host, the receptor-binding domain (RBD) of the S protein binds to cell receptors at the cell surface named angiotensin-converting enzyme II (ACE2).^11^ The region in the RBD that is directly involved in recognition between the RBD and ACE2 is termed the receptor-binding motif (RBM).^12–14^ Although most mutations occur in the RBM, there is still an opportunity for the identification of broad nmAbs recognizing some conserved regions in the RBD.^15^ By a single-B-cell-sorting method, we previously identified a human monoclonal antibody, 58G6, that could bind to RBDs from SARS-CoV-2 WT and the Alpha, Beta and Gamma variants.^9^ Here, we found that 58G6 retained its binding activity with EC_50_ values of 22.17 ng/ml for the Delta RBD and 22.50 ng/ml for SARS-CoV-2 WT RBD and still bound to the Omicron RBD, with an EC_50_ value that increased to 923.3 ng/ml and 953.7 ng/ml for Omicron BA.1 and BA.2, respectively (Fig. 1a).

Additionally, biolayer interferometry (BLI) was used to determine the binding affinities of 58G6 with the S proteins of WT, Delta, Omicron BA.1 and BA.2 SARS-CoV-2. 58G6 showed a strong affinity with Delta S and decreased avidity with Omicron BA.1 and BA.2 S proteins compared with WT, but a single-digit nanomolar affinity with Omicron BA.1 and BA.2 S proteins remained (Fig. 1d).

In our previous work, we found that two regions (450–458 and 470–495) in the RBD are involved in binding to 58G6.^9^ Several residues, including K458, Y473, S477, T478, E484, F486, N487 and Q493, in these two regions are part of the binding surface between 58G6 and the SARS-CoV-2 WT RBD and form hydrogen bonds. Among these residues, K458, Y473, F486, and N487 are conserved in SARS-CoV-WT and all VOCs, while K458, Y473, S477, F486, N487 and Q493 are conserved in SARS-CoV-WT and VOCs, excluding Omicron (Fig. 1e). In addition, 58G6 forms a hydrocarbon-hydrogen bond with the main chain of E484 in the RBD rather than the side chain, which allows it to invariably retain its neutralizing potency against the Beta variant.^9^ Hence, the mutation of residue 484 might not have a significant influence on binding. Together, the data indicate that 58G6 binds the Delta RBD similar to its binding of the WT SARS-CoV RBD, but binding to Omicron is weaker.

### 58G6 potently and broadly neutralizes different SARS-CoV-2 VOCs

The neutralizing activity of 58G6 was first evaluated using pseudotyped SARS-CoV-2 viruses, including the WT virus and the Delta and Omicron variants, based on the ability of 58G6 to bind a variety of RBDs (Fig. 1b). 58G6 showed broadly neutralizing activities against all tested pseudoviruses, exhibiting IC_50_ values ranging from 4.75 to 183.6 ng/ml. In a live viral plaque reduction assay, 58G6 neutralized authentic SARS-CoV-2 WT, Delta, and Omicron with IC_50_ values of 1.32 ng/ml, 1.69 ng/ml, and 54.31 ng/ml, respectively (Fig. 1c). Although we did not compare side by side, the IC_50_ values of 58 G against Omicron were much lower than those of many approved nAbs targeting RBM in clinical use because they almost lost neutralization activity against Omicron (IC_50_ > 10 μg/mL).^5–7^

### 58G6 delivered via intranasal administration shows protective efficacy against the Delta variant in vivo in a hamster model

**Fig. 2:**
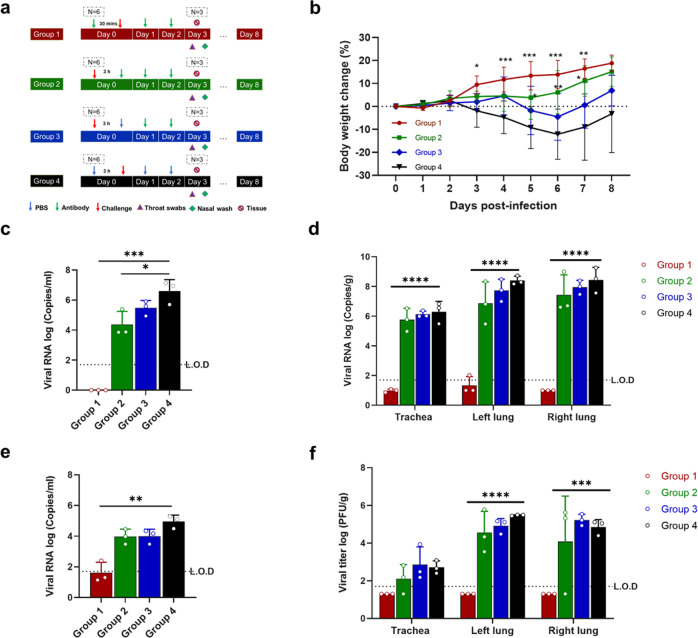
Intranasally delivered 58G6 offers protection against the Delta variant in a hamster model. **a** Animal experimental scheme. Hamsters were divided into 4 groups (*n* = 6 per group), including Group 1 (3-dose antibody treatment at 30 min pre-infection and 24 and 48 h post-infection), Group 2 (3-dose antibody treatment group at 3, 24, and 48 h post-infection), Group 3 (2-dose antibody treatment at 24 and 48 h post-infection) and Group 4 (control group administered PBS). **b** Body weight changes. During an eight-day observation period, the body weight changes of the hamsters in the control group and treatment groups were compared. **c** Viral RNA copies detected in the nasal washes of hamsters challenged with Delta at 3 dpi. **d** Viral RNA copies detected in the respiratory tracts of hamsters challenged with Delta at 3 dpi. **e** Viral RNA copies detected in the throat swabs of hamsters challenged with Delta at 3 dpi. **f** The number of infectious viruses (PFU) was measured in the respiratory tract at 3 dpi by a viral plaque assay in Vero E6 cells. Bars indicate the mean ± SD.

In our previous study, 58G6 exhibited protective efficacy in hACE2-transgenic mice (K18 mice) infected with SARS-CoV-2 WT and the beta variant when administered intraperitoneally. While K18 mice served as a very useful animal model for evaluating vaccines and therapeutics in the early stage of the pandemic, they are much more expensive, have more limited supplies and have recently been replaced/or partially replaced by golden (Syrian) hamsters. Several organs, including the lungs, liver, kidneys, and even the brain, can be severely damaged by SARS-CoV-2 in K18 mice.^16^ Therefore, this model might not be suitable for the evaluation of drugs administered nasally. SARS-CoV-2 can infect golden hamsters, resulting in the presence of viral antigens in the nasal mucosa and bronchial epithelial cells and lung consolidation.^17^ Experts in the field accept the golden hamster as a model for developing vaccines and therapies against SARS-CoV-2. Hence, we conducted an in vivo study with hamsters. In the prophylactic group (Group 1), hamsters were intranasally administered 5 mg/kg 58G6 30 min before challenge with SARS-CoV-2 Delta and given two more doses at 24 h and 48 h post-challenge (Fig. 2a). In the therapeutic efficacy assessment, the hamsters were intranasally administered 5 mg/kg 58G6 antibody (Group 2) or buffer only (Group 3) at 3 h post-infection. Then, these two groups were given two more doses of 58G6 at 24 h and 48 h post-infection. Another group (Group 4) was set as Group 1 but administered buffer only instead of antibody (Fig. 2a).

We monitored animal weight and found that the body weight in groups with antibody intervention (prophylaxis or treatment) either did not decrease or recovered faster than that in untreated groups (Fig. 2b). One-step real-time RT-PCR was used to assess viral RNA levels in the nasal wash (Fig. 2c), different tissue homogenates (Fig. 2d) and throat swab (Fig. 2e) at 3 days postinfection (dpi). Viral titers in tissue homogenates (Fig. 2f) were determined with a plaque assay. In general, the viral RNA levels in the nasal wash, all tested tissue homogenates, and throat swabs (Fig. 2c–e) were obviously lower in the prophylactic group (Group 1) than in the other groups. Consistently, the viral titer in the prophylactic group at 3 dpi was under the limit of detection (L.O.D.) (Fig. 2f).

### 58G6 reduces lung injury caused by Delta variant infection

**Fig. 3:**
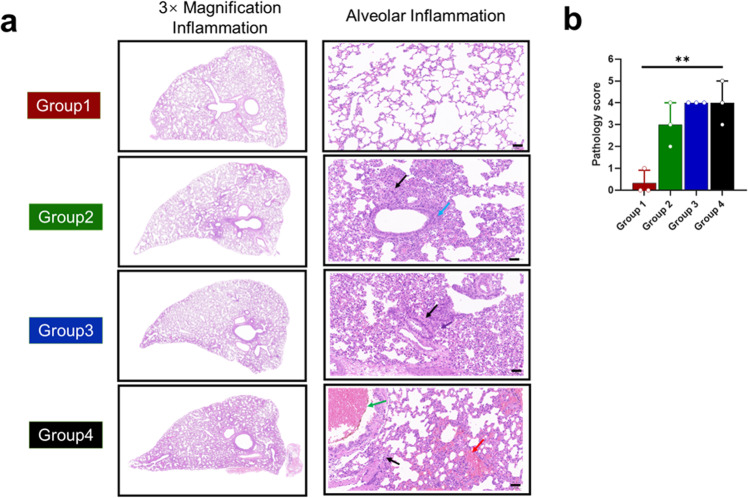
Histopathological changes in the lung at 3 dpi in hamsters challenged with the Delta variant. **a** Representative images of the H&E-stained lung tissues of Group 1, 2, 3, and 4 hamsters at 3 days after Delta variant challenge. There was no obvious abnormality in the structure of the bronchus at any level. The alveolar wall was composed of a single layer of epithelium with a clear structure. No obvious inflammatory changes were observed (Group 1, prophylactic group). Lung tissue showed small foci of alveolar wall thickening with a small amount of neutrophil infiltration (black arrows); vascular congestion and dilation were common (green arrows) (Group 2, 3-dose antibody treatment group). Lung tissue showed mild alveolar wall thickening with neutrophil infiltration (black arrows); occasionally, there were a few necrotic epithelial cells, lymphocytes, and neutrophils in the bronchi (purple arrows) (Group 3, 2-dose antibody treatment group). Diffuse hemorrhage in the alveolar wall (red arrow) was seen in the lung tissue; a large number of blood cells in the bronchi were often seen (green arrow); multifocal thickening of the alveolar wall with neutrophil infiltration (black arrow) was seen (Group 4, control group without antibody). **b** Cumulative histopathological score of the lung lesions post Delta variant infection in hamsters. The mean along with standard deviation is plotted on the graph.

We performed histological analysis on the lung samples from the three intervention groups and the control (buffer) group at 3 dpi (Fig. 3a). In the buffer group (Group 4), the lung samples showed increased infiltration of inflammatory cells around blood vessels and branches, extensive alveolar wall thickening, and a small amount of exudation after hematoxylin-eosin staining. In contrast, Group 1 (prophylactic group) did not show an increase in perivascular inflammatory cells, and Group 2 (3-dose 58G6 group) had a slightly increased number of perivascular inflammatory cells. In Group 3 (2-dose 58G6 group), a modest increase in the number of perivascular inflammatory cells and a slightly widened and thickened alveolar wall were observed. All sections were also pathologically scored for general evaluation of histological changes and inflammation progression (Fig. 3b). The prophylactic group (Group 1) showed a significantly reduced pathology score. One of the therapeutic groups (Group 2) also showed better outcomes, although the statistical significance was not very obvious. These results suggest that the nasal administration of 58G6 reduces lung pathology associated with SARS-CoV-2 infection.

### 58G6 delivered via intranasal administration also shows protective efficacy against the Omicron variant in vivo in a hamster model

**Fig. 4:**
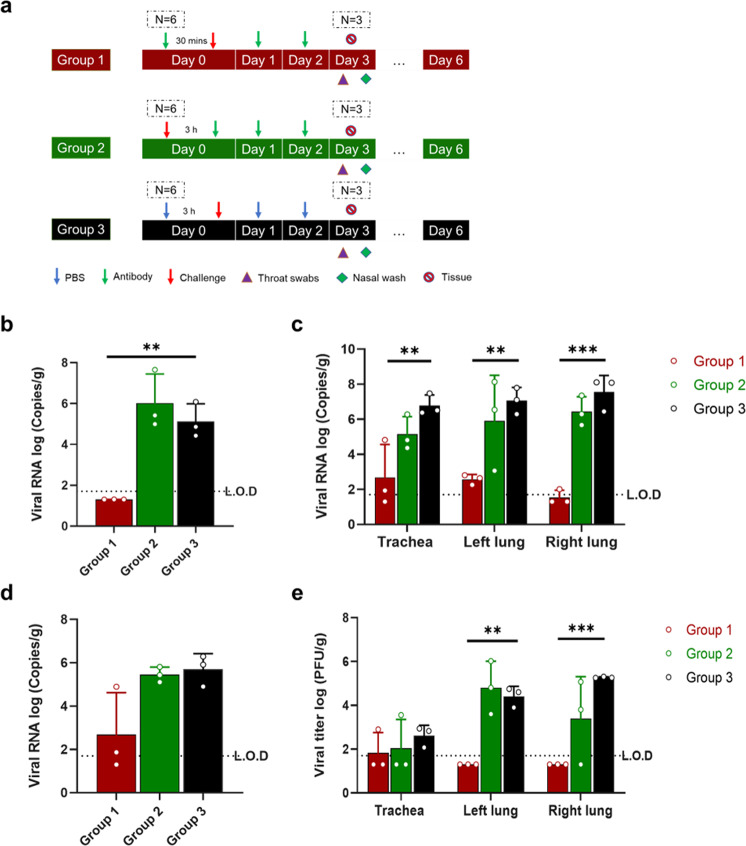
Intranasally delivered 58G6 offers protection against the Omicron variant in a hamster model. **a** Animal experimental scheme. Hamsters were divided into 3 groups (*n* = 3 per group), including Group 1 (3-dose antibody treatment at 30 min pre-infection and 24 and 48 h post-infection), Group 2 (3-dose antibody treatment group at 3, 24, and 48 h post-infection), and Group 3 (control group administered PBS). Viral RNA in the **b** nasal washes, **c** respiratory tracts and **d** throat swabs of hamsters challenged with the Omicron variant at 3 dpi. **e** The number of infectious viruses (PFUs) was measured in the respiratory tract at 3 dpi by the viral plaque assay in Vero E6 cells. The geometric mean along with the standard deviation is plotted on the graph.

The emergence of the SARS-CoV-2 Omicron variant seriously challenges the current nmAbs approved for clinical use and those under development. Hence, we also performed an animal study to evaluate the protective efficacy of 58G6 against Omicron (Fig. 4). The experiments were performed similarly to the procedure described for animals challenged by the Delta variant but with a 3-fold higher antibody dose and without the 2-dose 58G6 group (Fig. 4a). In general, 58G6 could also protect against Omicron variant challenge in hamsters. The viral RNA levels in the nasal wash, all tested tissue homogenates, and throat swabs (Fig. 4b–d) were significantly lower in the prophylactic group than in the other groups. Consistently, at 3 dpi, the viral titers in the prophylactic group were also significantly lower than those in other groups or under the L.O.D. (Fig. 4e). We did not observe an animal body weight decrease in any group (Supplementary Fig. S1), which was consistent with a recent report.^18^

**Fig. 5:**
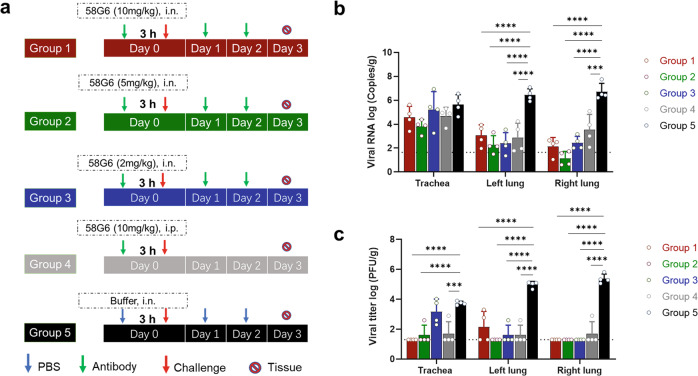
Determination of the prophylactic dose of 58G6 against Omicron in the hamster model. **a** Animal experimental scheme. Group 1, 10 mg/kg 58G6 (3-dose for i.n.); Group 2, 5 mg/kg 58G6 (3-dose for i.n.); Group 3, 2 mg/kg 58G6 (3-dose for i.n.); Group 4, 10 mg/kg 58G6 (1-dose for i.p.); Group 5, control group without 58G6. **b** Viral RNA copies in respiratory tracts of hamsters challenged with the Omicron variant at 3 dpi. **c** The number of infectious viruses (PFUs) was measured in the respiratory tract at 3 dpi by the viral plaque assay in Vero E6 cells. Bars indicate the mean ± SD.

We further performed experiments with three different doses of 58G6 (10, 5 and 2 mg/kg daily) as Groups 1, 2 and 3, respectively, via intranasal administration to evaluate prophylactic efficacies (Fig. 5a). In addition, a single dose of 58G6 (10 mg/kg) was administered intraperitoneally only once (Group 4), while a buffer group (Group 5) served as a control. All tested doses were effective (Fig. 5b, c). Notably, the viral RNA level and infectious virus titer in the 2 mg/kg dose group (Group 3) were generally similar to those in the other intervention groups. Compared to mock-infected controls, hamsters treated with 58G6 maintained a similar body weight increase (Supplementary Fig. S2). These data confirmed that 58G6 at an effective dose as low as 2 mg/kg reduced Omicron viral replication in the lungs and prevented disease symptoms without causing additional distress. Therefore, 58G6 could be an effective agent against the Omicron variant, although most nmAbs lose neutralizing activities against the Omicron variant, as mentioned above.

## Discussion

nmAbs are effective tools for combating infectious illnesses. Currently, transgenic mice, convalescent patients, and phage display have been used as sources to identify and characterize a panel of SARS-CoV-2 neutralizing antibodies. Notably, some of these antibodies have already been approved for the treatment of COVID-19. However, the protective efficacy of these mAbs against the two most dominant VOCs, Delta and Omicron, is either lost or significantly decreased.^5,6^ Among these approved mAbs, S309 (VIR-7831)^19^ maintained its neutralizing activity against Delta and Omicron in a pseudovirus assay.^5^ S309 (sotrovimab) is currently accepted for the treatment of mild-to-moderate COVID-19 under an Emergency Use Authorization (EUA). However, patients infected with SARS-CoV-2 who need hospitalization have not shown a benefit from treatment with sotrovimab. Therefore, broader nmAbs against SARS-CoV-2 that yield better clinical outcomes are still highly desired.

Our data indicate that 58G6 can efficiently neutralize authentic Delta and Omicron variants and inhibit viral infection in vivo when delivered through nasal administration. Importantly, 58G6 could provide prophylactic protection against Omicron even at a very low dose of 2 mg/kg. Hence, 58G6 is a good clinical candidate for use against infections caused by newly emerging SARS-CoV-2 variants (e.g., Delta and Omicron) in humans. In contrast with currently approved nmAbs, 58G6 could relieve patients’ burden due to the low dose used in animal studies and be more acceptable to different patients due to its convenient and safe nasal administration. More importantly, 58G6 is a broad nmAb that can inhibit SARS-CoV-2 and all currently recognized VOCs. Due to the conserved sites in the RBD recognized by 58G6, it is very possible that 58G6 is capable of neutralizing additional SARS-CoV-2 variants that emerge in the future.

## Materials and methods

### Ethics statements

Five- to six-week-old female Syrian golden hamsters were randomly assigned to different groups in this study. The biosafety level 3 (BSL-3) facility’s regular operating procedures were followed when performing viral infections. All procedures of the animal experiment were conducted in accordance with the approval for the care and use of laboratory animals by the Institutional Review Board of the Wuhan Institute of Virology, Chinese Academy of Sciences (ethics number: WIVA45202104).

### Cells and viruses

Vero E6 cells (catalog no. ATCC® CRL-1586™) were grown in Dulbecco’s modified Eagle’s medium (DMEM) (Gibco) with 10% FBS (Gibco), 100 units/ml penicillin, and 100 μg/ml streptomycin (Gibco) at 37 °C and with 5% CO_2_. The SARS-CoV-2 WIV04 (GISAID, accession no. EPI_ISL_402124) and Delta variant (B.1.617.2; GWHBEBW01000000) strains were originally isolated from COVID-19 patients and stored at the Wuhan Institute of Virology, Chinese Academy of Science. The SARS-CoV-2 Omicron virus (CCPM-B-V-049-2112-18) was isolated from a throat swab of a patient from Hong Kong by the Institute of Laboratory Animal Sciences, Chinese Academy of Medical Sciences. All studies with live SARS-CoV-2 in this study were performed in a BSL-3 facility.

### ELISA

SARS-CoV-2 RBD recombinant protein was coated onto ELISA plates (corning 96-well clear flat bottom, Cat. no. 9018) overnight at a concentration of 4 μg/ml in 0.05 M PBS, pH 9.6, 4 °C. Serially diluted 58G6 was added to each well and incubated for 1 h at 37 °C. Peroxidase-conjugated mouse anti-human IgG Fc antibody [HRP], mAb (GenScript, Clone ID 50B4A9) was used as the detection antibody. The absorbance at 450 nm was read in a microplate reader.

### Biolayer interferometry

Biolayer interferometry assays were carried out using the Octet R2 Protein Analysis System (Fortebio). Biotinylation and purification of 58G6 were performed using the EZ-link NHS-PEO Solid Phase Biotinylation Kit (Pierce) biotinylated and MINI Dialysis Unit (Thermo Fisher), respectively. The S proteins of SARS-CoV-2 and the Delta, Omicron BA.1 and Omicron BA.2 variants were immobilized onto AR2G biosensors (Fortebio) separately, with 58G6 used as an analyte to measure the affinities of 58G6 with four different sourced SARS-CoV-2 S proteins. After baseline adsorption of nonspecific binding, streptavidin (SA) biosensors (ForteBio) were immersed with biotinylated 58G6 to capture mAbs, and then the sensors were immersed in kinetics buffer (0.02% Tween-20, 1 mg/mL BSA in PBS) to the baseline. The disassociation was conducted after association with twofold diluted S proteins (diluted from 50 nM to 3.125 nM). Data were recorded using Octet BLI Discovery (12.0) and analyzed using Octet BLI Analysis (12.0).

### Inhibition of pseudotyped SARS-CoV-2 infection

These tests were carried out in accordance with a prior description.^9^ Briefly, 50 μl pseudoviruses (WT SARS-CoV-2, Delta, and Omicron) were incubated with serial dilutions of mAbs in a volume of 50 μl for 1 h at 37 °C and then added to ACE2-expressing Lenti-X293T cells (293 T/ACE2). The luciferase activities of infected 293T/ACE2 cells were measured 72 h after infection using the Bright-Luciferase Reporter Assay System (Promega, E2650), and IC_50_ values of the evaluated mAbs were determined with a Varioskan LUX Microplate Spectrophotometer (Thermo Fisher) and calculated by four-parameter logistic regression using GraphPad Prism 8.0.

### Neutralizing activities against authentic SARS-CoV-2

The details of an authentic SARS-CoV-2 neutralization assay performed in a BLS-3 laboratory have been described previously.^20^ In brief, Vero E6 cells were seeded at a density of 1.5 × 10^5^ cells per well in 24-well culture plates 1 day before the assay began. The antibody samples were serially diluted threefold in DMEM with 2.5% FBS in a volume of 200 μl. An equal volume including 120 PFU SARS-CoV-2 or variants was added, and the antibody-virus mixture was incubated at 37 °C for 1 h. Next, media from cells was removed, and 250 μl mixture was added to a 24-well culture plate containing Vero E6 cells. As positive and uninfected controls, cells with 75 PFU/250 μl SARS-CoV-2 alone and cells without the virus, respectively, were employed. After another incubation at 37 °C for 1 h, the antibody-virus mixture was taken out, and DMEM with 2.5% FBS plus 0.9% carboxymethyl cellulose was added to the cells. After culturing in a 37 °C incubator for 72 h, 8% paraformaldehyde was used to inactivate the virus and 0.5% percent crystal violet was used to stain the plates.

### Animal protection experiments

Female Syrian golden hamsters were challenged intranasally with 10^4^ PFU of the Delta and Omicron variants per animal in 100 μl of DMEM after anesthetization with isoflurane. For the prophylactic group (Group 1), hamsters were intranasally administered 100 μl of 58G6 (5 mg/kg for Delta challenge and 15 mg/kg for Omicron challenge) 30 min before challenge and given two more doses at 24 h and 48 h post-challenge. The activity of 58G6 was also assessed in an in vivo therapeutic experiment. Alternatively, hamsters were intranasally administered 100 μl of 5 mg/kg (Delta challenge) or 15 mg/kg (Omicron challenge) 58G6 antibody (Group 2) or PBS (Group 3, this group was established only in the Delta challenge but not the omicron challenge) at 3 h post-challenge, followed by two doses of 100 μl of 5 mg/kg (Delta challenge) or 15 mg/kg (Omicron challenge) 58G6 antibody at 24 h and 48 h post-challenge. The negative control group (Group 4; this group was named Group 3 in the Omicron challenge) was treated the same as Groups 2 and 3 but with the equivalent volume of PBS instead of antibody. At 3 dpi, three animals were euthanized, and nasal washes, throat swabs and tissues (lung and trachea) were harvested for analysis. The remaining three hamsters were monitored for weight loss until 8 dpi in the protection experiment against Delta or 6 dpi in the protection experiment against Omicron.

After the prophylactic dose of 15 mg/kg was tested, we further decreased the dose of 58G6 to three groups, 10 mg/kg (Group 1), 5 mg/kg (Group 2), and 2 mg/kg (Group 3), to evaluate the prophylactic efficacy against Omicron. In general, the experiments were performed as mentioned above. Here, hamsters were intranasally administered 58G6 3 h before challenge and given two more doses at 24 h and 48 h post-challenge. In addition, we set a group (Group 4) with only one dose (10 mg/kg) of 58G6 via intraperitoneal administration 3 h before challenge. The buffer group (Group 5) was used as a control. All the samples were collected and analyzed.

### Virus RNA copy numbers and titers

We quantified viral RNA in nasal washes, throat swabs, and tissue homogenates by one-step real-time RT-PCR as described in the previous article.^21^ We purified viral RNA using the QIAamp Viral RNA Mini Kit from Qiagen, and performed RT-qPCR detection with the HiScript II One-Step qRT–PCR SYBR® Green Kit from Vazyme Biotech Co., Ltd. qRT–PCR was carried out using primers ORF1ab-F (5’-CCCTGTGGGTTTTACACTTAA-3’) and ORF1ab-R (5’-ACGATTGTGCATCAGCTGA-3’) under 50 °C for 3 min, 95 °C for 30 s, and 40 cycles of 95 °C for 10 s and 60 °C for 30 s. A plaque assay was used to determine virus titer, slightly modified from that described previously.^22^ Briefly, approximately 1.5 × 10^5^ Vero E6 cells/well were prepared into 24-well plates, and the plate was incubated overnight at 37 °C. The sample supernatant was serially diluted with DMEM with 2.5% FBS from 10^−1^ to 10^−4^ by 10-fold dilution and inoculated with Vero E6 cells at 37 °C in 5% CO_2_ for 1 h. Then, the inoculum was removed, and DMEM containing 2.5% FBS and 0.9% carboxymethyl-cellulose was added. After incubation at 37 °C in 5% CO_2_ for 3 days, the viral titer was calculated by counting plaques that had been fixed in 8% paraformaldehyde and stained in 0.5% crystal violet.

### Histological analysis

Lung tissue was fixed with 10% neutral formaldehyde and stained with hematoxylin and eosin (HE). Histologic lesion severity was scored per lung lobe according to a standardized scoring system by evaluating the area percentages of alveolar wall thickening, type II pneumocyte hyperplasia, edema and fibrin, perivascular lymphoid cuffing, hemorrhage and inflammatory cell infiltration: 0, no lesions; 1, minimal (1–10% of lobe affected); 2, mild (11–25%); 3, moderate (26–50%); 4, marked (51–75%); 5, severe (76–100%).

### Statistical analysis

GraphPad Prism 9 was used for all statistical analyses. For comparisons between two groups, an unpaired t test followed by Welch’s correction for unequal standard deviations was employed. The asterisks shown in the figures refer to the level of significance: *p ≤ 0.05; **p ≤ 0.01; ***p ≤ 0.001; ****p ≤ 0.0001.

## Supplementary information

Supplementary Materials

## Data Availability

All other data are available from the corresponding author upon reasonable request. Source data are provided with this paper.
