# Supplementary material for: A potent neutralizing antibody provides protection against SARS-CoV-2 Omicron and Delta variants via nasal delivery
Source: Signal Transduct Target Ther. 2022 Aug 30;7:301. doi: 10.1038/s41392-022-01135-3 (PMC9424799; doi:10.1038/s41392-022-01135-3)
Supplement: Supplementary file 1 — Supplementary Materials [file 41392_2022_1135_MOESM1_ESM.docx]

Supplementary Materials for

A potent neutralizing antibody that provides protection against SARS-CoV-2 Omicron and Delta variants via nasal delivery

Xinghai Zhang^1†^, Huajun Zhang^1†^, Tingting Li^2,3†^, Shaohong Chen^1,4†^, Feiyang Luo^2,3†^, Junhui Zhou^1,4†^, Peiyi Zheng^5^, Shuyi Song^2,3^, Yan Wu^1^, Tengchuan Jin^5^, Ni Tang^6^, Aishun Jin^2,3^, Chengyong Yang^7^, Guofeng Cheng^7*^, Rui Gong^1*^, Sandra Chiu^5*^, Ailong Huang^6*^

^*^Correspondence to: Guofeng Cheng, guofeng.cheng@ausperbio.com; Rui Gong, gongr@wh.iov.cn; Sandra Chiu, qiux@ustc.edu.cn; and Ailong Huang, ahuang@cqmu.edu.cn.

**This PDF file includes:**

Figure. S1-S2

**
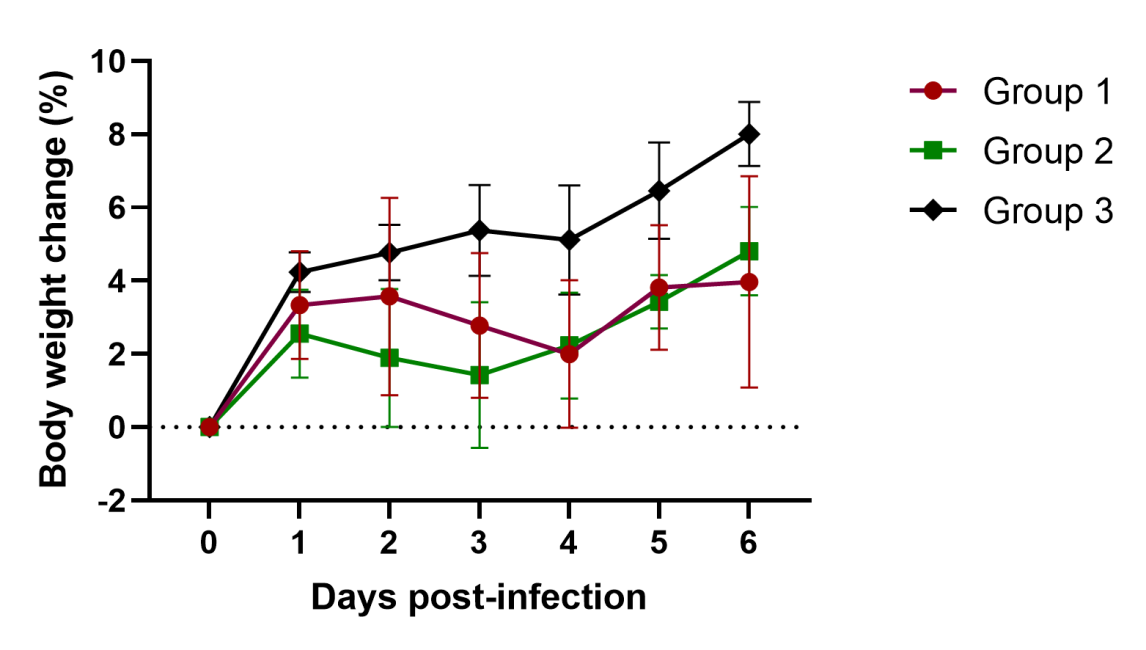
**

**Figure. S1.** Body weight change in the experiment of protective efficacy of 58G6 against Omicron in the hamster model. Group 1, prophylactic group; Group 2, treatment group; Group 3, control group without antibody.

**
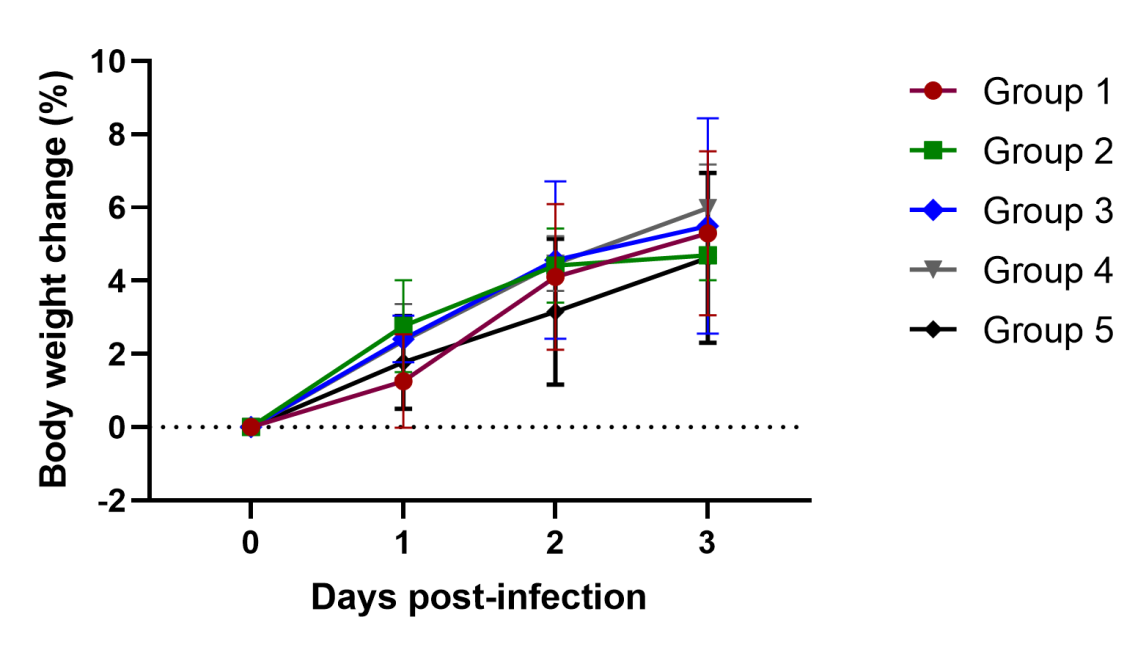
**

**Figure. S2.** Body weight change in the experiment of determination of prophylactic dose of 58G6 against Omicron in the hamster model. Group 1, 10 mg/kg 58G6 (3-dose for i.n); Group 2, 5 mg/kg 58G6 (3-dose for i.n); Group 3, 2 mg/kg 58G6 (3-dose for i.n); Group 4, 10 mg/kg 58G6 (1-dose for i.p); Group 5, control group without 58G6.
